# Effect of Ammonia and Indole-3-acetic Acid Producing Endophytic *Klebsiella pneumoniae* YNA12 as a Bio-Herbicide for Weed Inhibition: Special Reference with Evening Primroses

**DOI:** 10.3390/plants9060761

**Published:** 2020-06-18

**Authors:** Sang-Mo Kang, Saqib Bilal, Raheem Shahzad, Yu-Na Kim, Chang-Wook Park, Ko-Eun Lee, Jeong-Ran Lee, In-Jung Lee

**Affiliations:** 1 Institute of Agricultural Science and Technology, Kyungpook National University, Daegu 41566, Korea; sangmo@knu.ac.kr; 2 School of Applied Biosciences, Kyungpook National University, Daegu 41566, Korea; dbsk7622@knu.ac.kr (Y.-N.K.); wcp423@naver.com (C.-W.P.); ge8340@hanmail.net (K.-E.L.); 3 Natural and Medical Sciences Research Center, University of Nizwa, Nizwa 616, Oman; saqib@unizwa.edu.om; 4 Basic and Applied Scientific Research Center, Imam Abdulrahman Bin Faisal University, Dammam 31441, Saudi Arabia; rmshahzad@iau.edu.sa; 5 Department of Biology, College of Science, Imam Abdulrahman Bin Faisal University, Dammam 31441, Saudi Arabia; 6 Crop Protection Division, National Institute of Agricultural Science, RDA, Wanju 55365, Korea; kongsarang@korea.kr

**Keywords:** endophyte, IAA, bio-herbicide, abscisic acid, catalase, macronutrients

## Abstract

Information on the use of endophytic bacteria as a bio-herbicide for the management of weed control in agricultural fields is limited. The current study aimed to isolate endophytic bacteria from evening primroses and to screen them for their bio-herbicidal activity. Two isolated endophytic bacteria (*Pantoea dispersa* YNA11 and *Klebsiella pneumoniae* YNA12) were initially screened for citrate utilization and for indole-3-acetic acid (IAA) and catalase production. The preliminary biochemical assessment showed YNA12 as a positive strain. Ammonia, catalase, and IAA in its culture filtrate were quantified. Gas Chromatography/Mass Spectroscopy- Selective Ion Monitoring (GC/MS-SIM) analysis revealed the production of IAA by YNA12 in a time-dependent manner. YNA12 also exhibited significant ammonia-producing potential and catalase activity against hydrogen peroxide. The YNA12 culture filtrate significantly inhibited the germination rate of evening primrose seeds, resulting in a marked reduction in seedling length and biomass compared with those of the control seeds. Moreover, the culture filtrate of YNA12 significantly accelerated the endogenous abscisic acid (ABA) production and catalase activity of evening primrose seedlings. Macronutrient regulation was adversely affected in the seedlings exposed to the culture filtrate of YNA12, leading to inhibition of seed germination. The current results suggest that endophytic YNA12 may be used as a potent bio-herbicidal agent for controlling weed growth and development.

## 1. Introduction

Plants interact with various microbes (phytopathogenic and phytobeneficial) throughout their life cycle in a soil ecosystem [1]. This interaction widely influences plant growth, development, and productivity [2]. Each microorganism has different direct and indirect strategies to influence plant growth and function by actively colonizing host plant tissues, providing microbial compounds to the host plant, facilitating the uptake of nutrients, and reducing and preventing the harmful effects of phytopathogens [3,4]. However, some microorganisms are known as deleterious microorganisms, which can be defined as microorganisms that inhibit plant growth without causing any apparent disease [5]. 

Deleterious microorganisms inhibit plant growth via several mechanisms [6,7], which include the production of phytotoxins such as cyanide as well as other volatile and nonvolatile compounds [8,9]. However, plant growth inhibition through phytohormone production is also thought to be an alternative mechanism of this group of microbes. Indole-3-acetic acid (IAA) produced by deleterious bacteria has been reported to inhibit root growth in various crops such as spiny amaranth (*Amaranthus spinosus*)*,* purslane (*Portulaca oleracea*), sugar beet, and blackcurrant [8]. These bacteria may also compete with plants and beneficial microorganisms for nutrients, which would contribute to the reduction of plant growth. Moreover, the bacteria may also indirectly inhibit plant growth by reducing the effect of nitrogen-fixing bacteria [6].

Conventional agriculture mostly relies on the use of heavy chemicals for higher agriculture productivity; however, the widespread use of chemicals in agriculture could threaten food safety [10,11,12]. In such situations, the use of phytohormone-producing microbial inoculants could be an ideal eco-friendly approach not only for promoting host plant growth and inhibiting weed growth but also for minimizing chemical input in agricultural systems [13]. Therefore, to produce safe agricultural products with eco-friendly methods and to minimize the use of chemical fungicides, pesticides, herbicides, and plant growth retardants, the use of plant-associated microbes may be considered to control pests and to retard the growth of unwanted plants [14]. 

Many microorganisms are used to improve plant growth and productivity via symbiotic interactions [15]. However, the use of microorganisms for plant growth inhibition has not been fully explored. Microorganisms that inhibit plant growth are needed for the inhibition of unwanted plants in agricultural fields with minimum or no chemical input. Among the different microorganisms, seed-borne endophytic bacteria are the most promising due to various phytobeneficial traits including phytohormone production, active colonization, plant growth promotion, and biotic and abiotic stress mitigation [3]. Evening primrose (*Oenothera biennis*) greatly affects the domestic ecosystem in South Korea due to rapid germination and seed production. However, studies are lacking regarding the management of *Oenothera biennis* that has rapidly spread nationwide and is disturbing domestic ecosystems. Therefore, the current study investigated the potential of microbial utilization by isolating seed-borne endophytic bacteria acting as biocontrol agent (BCA) in order to inhibit the growth of rapidly spreading evening primrose growth by hormone and enzyme secretion. In addition, the physiological basis of this phenomenon was evaluated by examining the regulation of endogenous phytohormones, antioxidants, and macronutrients in the host plant.

## 2. Materials and Methods

### 2.1. Isolation and Identification of Endophytic Bacteria

The seeds of local evening primrose (*Oenothera biennis*) were obtained from a farm in Gunwi in South Korea. The seeds were thoroughly rinsed with autoclaved double-distilled water and surface-sterilized by immersion in sodium hypochlorite (NaOCl) solution containing 2.5% active chloride (wt/vol) and 1 droplet of Tween 80 per 100 mL solution. To ensure the efficiency of the sterilization process, randomly selected seeds and the last few drops of the final rinse were incubated on Luria-Bertani LB medium at 30 °C for 3 days. Thereafter, approximately 30 seeds were carefully macerated in a sterilized phosphate buffer saline solution and serially diluted up to 10^−3^ dilution. From the diluted solution, 0.2 mL increments were plated on LB media plates and incubated at 27 °C for 48 h for the isolation of endophytic bacterial strains.

Thereafter, the newly endophytic bacterial isolates YNA11 and YNA12 were chosen due to their metabolite-producing potential among the 20 isolated strains. The preliminary biochemical tests and, thereafter, IAA and ammonia quantification of the selected bacterial isolates YNA11 and YNA12 were done through using Gas Chromatography-Mass Spectroscopy- Selective Ion Monitoring GC-MS/SIM and MI405 Ammonia Medium Range Photometer, respectively. Moreover, YNA11 and YNA12 were grown on LB medium at 30 °C for 3 days, and the bacterial pellet was extracted from the culture medium by centrifugation. The obtained pellet was further used for the extraction of genomic DNA to identify the endophytic YNA11 and YNA12 isolates by amplification and sequencing of the 16S rRNA region as described by Kang et al. [16], using the primer pair 27F (5′-AGA GTT TGA TCC TGG CTC AG-3′) and 1492R (5′-TAC CTT GTT ACG ACT T-3′). The resulting 16S rRNA sequence was then BLASTed against the National Center for Biotechnology Information (NCBI) GenBank database to investigate the nucleotide sequence homology of the isolated strains, and phylogenetic analysis was conducted using the neighbor-joining method with MEGA v. 7.0 [17]. The 16S rRNA sequences of *Pantoea dispersa* YNA11 and *Klebsiella pneumoniae* YNA12 were deposited to the NCBI GenBank database. 

### 2.2. Preliminary Biochemical Assessment of Endophytic Bacteria

#### 2.2.1. IAA Detection

Preliminary screening was performed to detect the IAA-producing ability of endophytic YNA11 and YNA12 by Salkowski’s test as described by Kang et al. [18]. In brief, equal volumes (0.5 mL) of the bacterial cell-free supernatant of 24-h-old cells grown in LB medium and Salkowski’s reagent were mixed together and kept in the dark at room temperature for 30 min. The development of pink coloration after 30 min was regarded as a positive indicator for the production of IAA.

#### 2.2.2. Citrate Utilization Test

Citrate utilization test was conducted as described by MacWilliams [19] by streaking endophytic YNA11 and YNA12 on Simmons citrate agar plate containing per L of distilled water, NaCl (5.0 g), sodium citrate (2.0 g), ammonium dihydrogen phosphate (1.0 g), magnesium sulfate (0.2 g), dipotassium phosphate (1.0 g), bromothymol blue (0.08 g), and agar (15.0 g). Then, the plates were incubated at 28.5 °C for 24 h. 

#### 2.2.3. Catalase Test

The catalase test of endophytic YNA11 and YNA12 was conducted as described by Tiwari and Singh [20]. In brief, 24-h-old bacterial cells were transferred to a clean slide using an inoculation loop and were exposed to several drops of 3% hydrogen peroxide (H_2_O_2_). The formation of oxygen bubbles indicated a positive test for the production of catalase by endophytic bacteria. 

### 2.3. Biochemical Analysis 

#### 2.3.1. Quantification of IAA Production by GC/MS-SIM

After IAA production was detected based on the preliminary Salkowski’s test, the culture filtrate of YNA12 (24 h, 48 h, and 72 h old grown in LB medium) was further used for the quantitation of IAA via GC-MS/SIM (5973 Network Mass Selective Detector and 6890N Network Gas Chromatograph; Agilent Technologies, Palo Alto, CA, USA) as described by Kang et al. [18]. In brief, 20 mL of the bacterial culture was centrifuged at 10,000× *g* for 15 min at 4 °C and the supernatant was filtered through a 0.45-µm cellulose membrane acetate. The resulting filtrate was acidified to pH 2.8 and supplemented with an internal standard of 40 μL of [D5]-IAA followed by extraction with 4 mL of ethyl acetate. The ethyl acetate extract was rotary evaporated under vacuum at 40 °C. The organic layer was vacuum-dried and amended with 60% methanol followed by adjustment to pH 8.0 ± 0.3 and by passing through a reverse-phase C18 column. The extracted residue was dissolved in 1.0 mL of methanol and 1.5 mL of ethereal diazomethane. Ethyl acetate was used for redissolving the methylated samples before the quantification of IAA by GC-MS/SIM.

#### 2.3.2. Production of Ammonia 

The production of ammonia by YNA12 was investigated as described by Lee et al. [21]. In brief, 100 µL of the culture broth of YNA12 grown in LB medium was transferred to tubes and diluted 100-fold in distilled water followed by the addition of Nessler’s reagent (0.5 mL). LB broth was used as the control. The value was measured using MI405 Ammonia Medium Range Photometer (range: 0.00 to 9.99 mg L^−1^; Milwaukee, Szeged, Hungary).

#### 2.3.3. Catalase Activity 

The catalase activity of bacterial cells and seedlings was determined by using the Amplex Red Catalase Assay Kit (Thermo Fisher, Waltham, MA, USA) as described by Park et al. [22]. In brief, 24-h, 48-h, and 72-h bacterial cultures (optical density (O.D_600_) = 0.2) were centrifuged, and the pellets were lysed in 0.1 M Tris buffer (pH 7.4). The experiment was conducted in a 96-well plate by adding 25 μL YNA12 cell lysates, catalase standard, and 40 µM H_2_O_2_. Subsequently, 50 μM Amplex Red reagent and 0.2 U/mL horseradish peroxidase were added. The absorbance was measured at 590 nm using a Multiskan™ GO microplate spectrophotometer (Thermo Fisher, Waltham, MA, USA).

### 2.4. Screening of Plant Growth Inhibition by Endophytic Bacteria via Germination Test

The seeds of evening primrose were surface-sterilized with 5% NaOCl followed by thoroughly rinsing with double-distilled water, and they were kept in Petri dishes containing two layers of filter paper. The culture broth (72 h old) of the isolated endophytic bacteria YNA12 was diluted (100 ppm) and added to the Petri dishes containing 30 evening primrose seeds. The Petri dishes were incubated at 28 °C for 7 days in an aseptic environment. LB medium (100 ppm) was used as a negative control, while the combined application of YNA12 culture filtrate, ammonia (10 ppm) and IAA (100 ppm) was used as a positive control. The following treatments were included in the experimental design: (i) control seeds treated with LB medium; (ii) ammonia-treated seeds; (iii) IAA-treated seeds; (iv) YNA12-culture broth-treated seeds; and (v) ammonia-, IAA-, and YNA12-culture broth-treated seeds. The bio-herbicide effect of endophytic YNA12 was assessed by measuring the seed germination rate, seedling length and weight, as well as cotyledon development frequency (CDF).

#### 2.4.1. Effect of YNA12 on the Abscisic Acid (ABA) Modulation of Evening Primroses

Endogenous ABA was extracted from the germinated seedlings of evening primroses as described by Qi et al. [23], with slight modifications as reported by Bilal et al. [24]. In brief, freeze-dried samples of evening primrose seeds were extracted with isopropanol (95%) and glacial acetic acid (5%) followed by supplementation with 20 ng of the internal standard Me-[^2^H_6_]-ABA. The resulting filtrate was vacuum-dried using a rotary evaporator, dissolved in 1 N NaOH, and washed with methylene chloride, and the pH was adjusted to 3.5. The obtained extract was dissolved in dichloromethane followed by transferring to a silica cartridge. Then, diethyl ether and methanol (3:2, v/v) were added to elude ABA from the cartridge. The resulting extract was dried using N_2_ gas and was methylated by adding diazomethane for the detection and quantification of ABA using GC-MS coupled with a selected ion monitor (SIM; 5973 Network Mass Selective Detector and 6890 N Network Gas Chromatograph System; Agilent Technologies, Palo Alto, CA, USA). The Lab-Base (ThermoQuest, Manchester, UK) data system software was used to monitor response signals to ions of m/e 162 and 190 for Me-ABA and 166 and 194 for Me-[^2^H_6_]-ABA.

#### 2.4.2. Analysis of Macronutrient Regulation in Plants by Inductively Coupled Plasma Mass Spectrometry (ICP-MS)

The freeze-dried powdered samples of evening primroses were used for the quantification of macronutrients (Potassium, K; Phosphorus, P; Calcium, Ca) by ICP-MS (Optima 7900DV; Perkin-Elmer, Waltham, MA, USA), as previously described by Bilal et al. [25]. In brief, 0.05 g of the ground plant sample was digested with 7 mL of 65% HNO_3_ and 1 mL of 30% H_2_O_2_ and subjected to microwave digestion at 180 °C for 20 min. Then, the sample was cooled for 40 min and quantified by ICP-MS. 

### 2.5. Statistical Analysis

All of the experiments were repeated three times, and the data collected from each repetition were pooled together. The current data illustrate the mean values with standard deviation (SD). The means were analyzed to find the significant differences among treatments by using one-way analysis of variance (ANOVA), followed by Duncan’s multiple range test (DMRT) in SAS software (V9.1, Cary, NC, USA).

## 3. Results

### 3.1. Identification and Phylogenetic Analysis of YNA11 and YNA12

**Figure 1:**
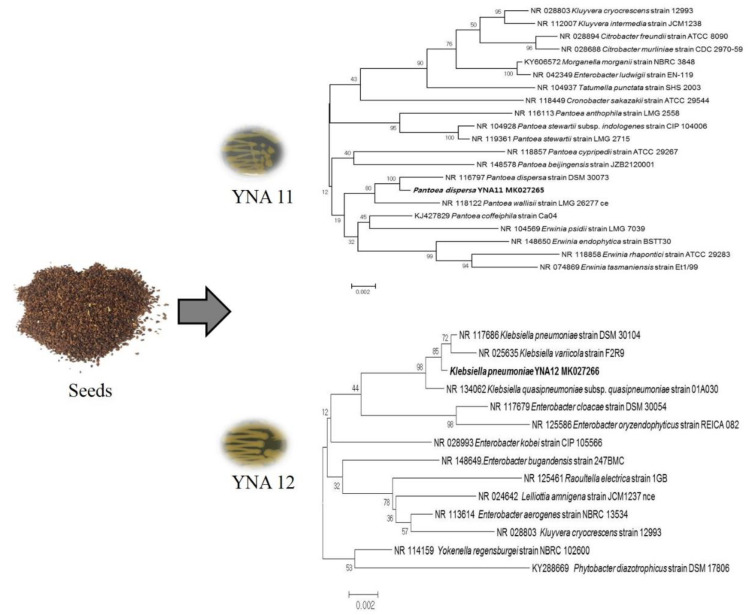
Neighbor-joining (NJ) phylogenetic tree constructed using the 16S rRNA sequences from endophytic YNA11 and YNA12 and related taxa.

For molecular identification and phylogenetic analysis of YNA11 and YNA12 isolates, 16S rRNA was amplified, sequenced, and compared to the database of known 16S rRNA genes. The sequences of different genera were also used for constructing the phylogenetic tree to determine the relationship among participating candidates in the group. The amplification of the 16S rRNA sequence and further phylogenetic analysis of YNA11 and YNA12 revealed that the isolated strains have maximum sequence homology with *Pantoea dispersa* and *Klebsiella pneumoniae*, respectively (Figure 1). The 16S rRNA sequences of *Pantoea dispersa* YNA11 and *Klebsiella pneumoniae* YNA12 were deposited to the NCBI GenBank database under the accession numbers MK027265 and MK027266, respectively. 

### 3.2. Biochemical Characterization

**Figure 2:**
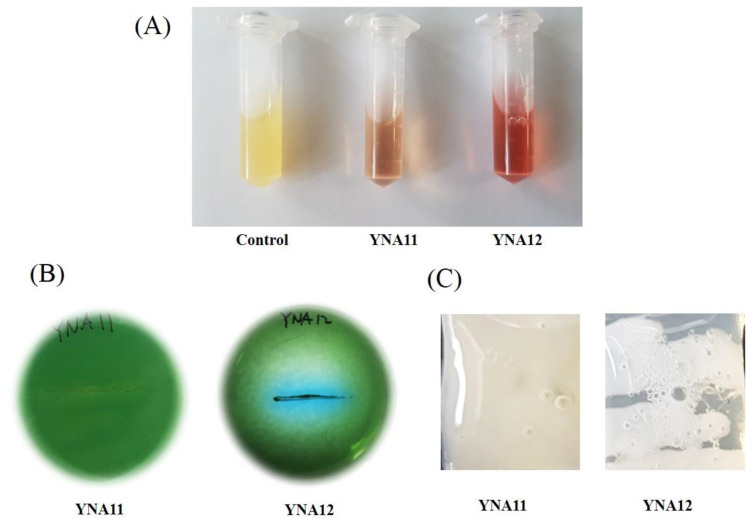
Preliminary biochemical screening of endophytic YNA11 and YNA12: (**A**) Salkowski’s test, (**B**) citrate utilization test, and (**C**) catalase test.

The preliminary biochemical assessment of the isolated endophytic bacteria revealed YNA12 as a positive strain with the ability to use citrate as a carbon source, indicated by a change in the color of the bromothymol blue indicator from green to blue (Figure 2B). YNA12 also reacted positively to the catalase test by producing catalase enzymes that catalyze the release of oxygen bubbles, thus protecting itself from the hazardous effects of H_2_O_2_ (Figure 2C). On the other hand, YNA11 was negative for citrate utilization and catalase production in the preliminary biochemical assessment. Similarly, Salkowski’s reagent test was carried out to confirm IAA production by endophytic YNA12 and YNA11. The results showed the appearance of a pink color following the addition of the Salkowski’s reagent to the culture filtrate of YNA12, indicating the production of IAA by endophytic YNA12 (Figure 2A). In contrast, no pink color was observed in the culture filtrate of YNA11 following the addition of Salkowski’s reagent.

### 3.3. Quantification of Metabolite Production by YNA12

**Figure 3:**
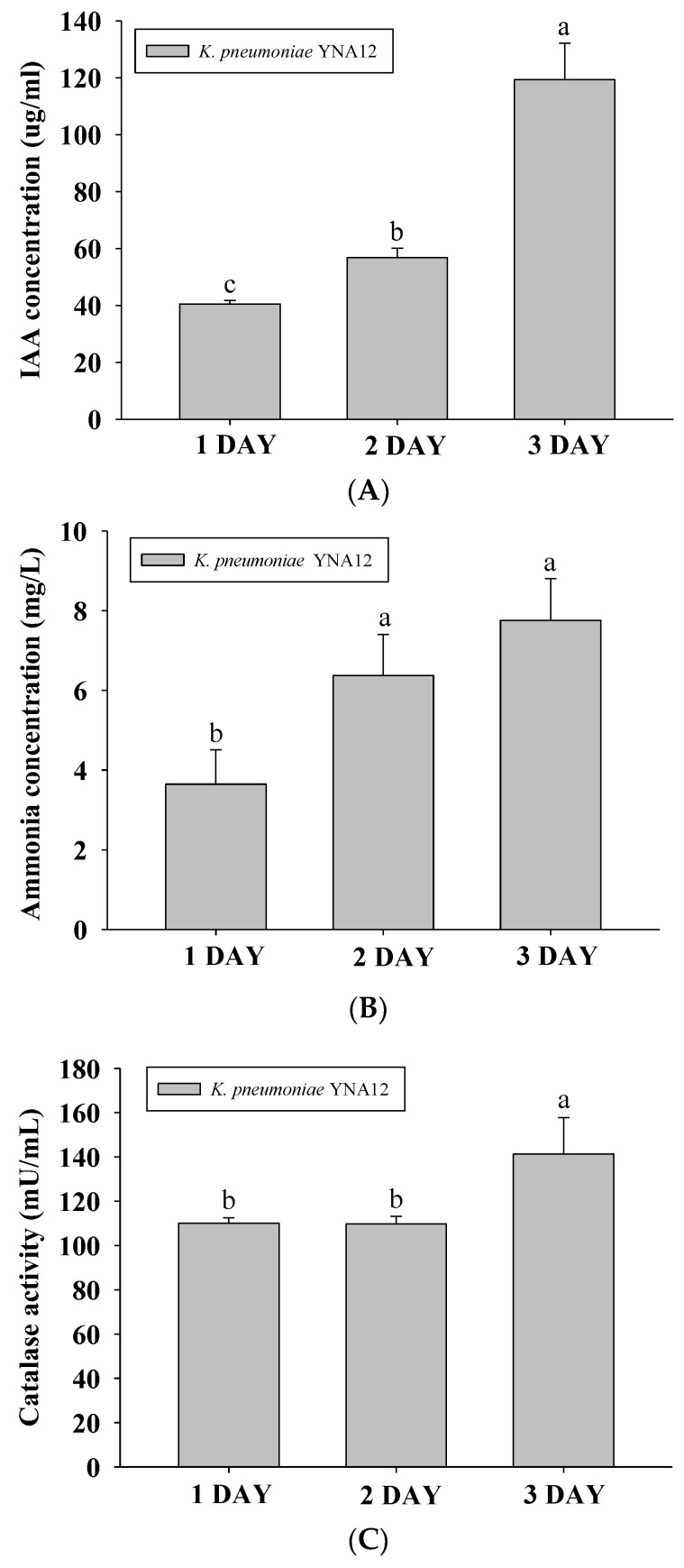
Quantification of indole-3-acetic acid (IAA) (**A**), ammonia (**B**), and catalase (**C**) produced by endophytic YNA12: Values and error bars represent the mean ± standard deviation (*n* = 3). Means followed by the same letter (a, b, and c) are not significantly different (*p* ≤ 0.05) according to Duncan’s multiple range test.

After the positive results of the preliminary biochemical tests, the culture filtrate of YNA12 was used for further analysis by GC/MS for the quantification of specific indole compounds. A higher level of IAA was detected in the 3-day-old culture medium of YNA12, followed by the 2-day-old and 1-day-old culture filtrates. The highest level of IAA in the 3-day-old culture filtrate of YNA12 was 119.3 ± 10.4 µg/mL (Figure 3A). Moreover, assessment of ammonia production by YNA12 revealed that the highest level of ammonia was detected in the 2-day-old and 3-day-old culture filtrates, followed by the 1-day-old culture filtrate. The highest level of ammonia was 7.75 ± 1.0 mg/L (Figure 3B). In the preliminary test for catalase production, YNA12 exhibited a positive result for the catalase test by producing oxygen bubbles. Therefore, the quantification of catalase production in response to H_2_O_2_ toxicity demonstrated a high tolerance of YNA12 against oxidative stress. Catalase production by YNA12 was significantly higher in the 3-day-old cell lysate, followed by the 1-day-old and 2-day-old cell lysates (Figure 3C).

### 3.4. Adverse Effects of Endophytic YNA12 on Seed Germination of Evening Primrose

The effect of the culture filtrate of YNA12 on the germination of evening primrose seeds was examined to determine the inhibition of seed germination at two time points, i.e., 7 days after treatment (DAT) and 5 DAT (Table 1). In addition, the germination rate of evening primrose seeds was significantly enhanced at 7 DAT compared with 5 DAT in all treatments. The application of a sole culture broth of YNA12 as well as the coupled application of ammonia, IAA, and YNA12 at 5 DAT equally resulted in a significantly lower germination rate compared with the control, the sole IAA-treated, and the sole ammonia-treated plants. The findings illustrated that application of a combined treatment of culture broth of YNA12, IAA, and ammonia exhibited approximately 9%, 37%, 65%, and 73% lower germination rates as compared to sole YNA12 culture broth, sole ammonia, sole IAA, and control seeds, respectively, at 5 DAT. Similarly, the germination rate at 7 DAT was noted to be significantly lowered in the combined application of YNA12 culture broth, IAA, and ammonia, followed by sole YNA12 culture broth, sole ammonia, sole IAA, and control seeds, respectively, whereas the combined application of YNA12 culture broth IAA and ammonia led to a significant deterioration of the seedling length and weight. Our results showed that the inhibition of the seed germination rate at 5 DAT with the combined application of YNA12 culture broth IAA and ammonia broth significantly decreased the seedling lengths by 33%, 65%, 76%, and 87% as compared with those of sole YNA12 culture broth, sole ammonia, sole IAA, and control seeds, respectively. Likewise, treatment of the culture broth of YNA12 and the combined application of YNA12 culture broth IAA and ammonia resulted equally in significant reductions of seedling length at 7 DAT, followed by sole ammonia, sole IAA, and control seedlings, respectively. Moreover, the seedling weight was drastically influenced by the application of sole YNA12 culture broth and the combined application of YNA12 culture broth, sole ammonia, and IAA by significantly reducing the seedling weight at 5 DAT as compared to those of sole ammonia, IAA, and control seedlings, respectively. However, a reduction of seedling weight at 7 DAT was not significant among combined treated seedlings, sole YNA12 culture broth-treated seedlings, and sole ammonia-treated seedlings. Similarly, Cotyledon development frequency (CDF) at 5 DAT and 7 DAT showed significant reduction under the combined YNA12 culture broth and the sole ammonia and IAA applications, followed by those of sole YNA 12 culture broth, sole ammonia, and sole IAA-treated seedlings as compared to control seedlings, respectively.

### 3.5. Effect of YNA12 on the Catalase Activity of Evening Primroses

**Figure 4:**
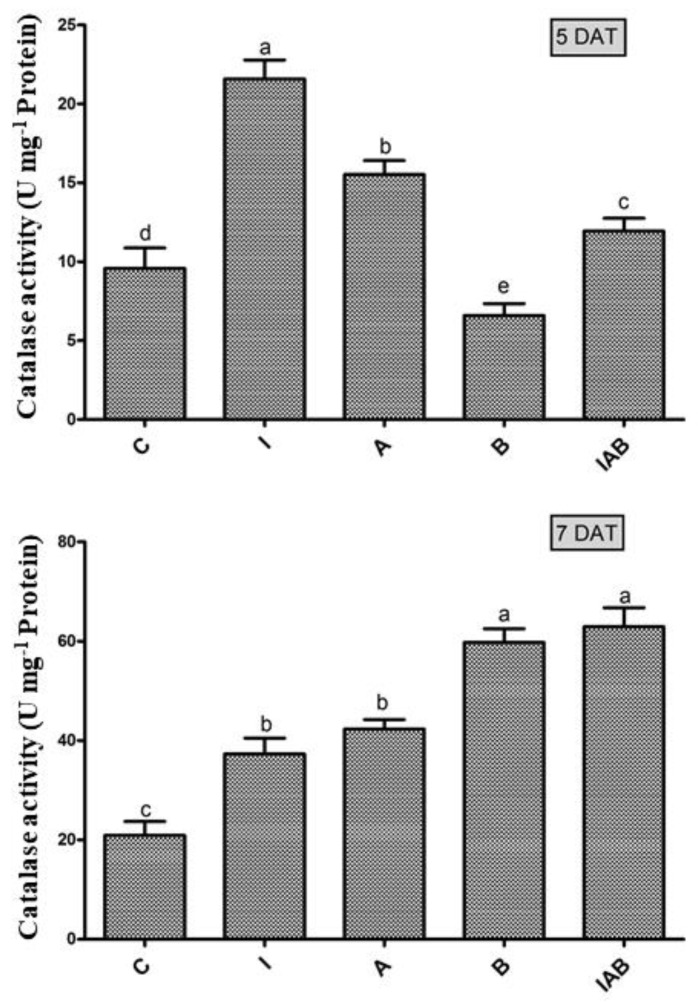
Effect of the YNA12 culture filtrate on the regulation of catalase in evening primrose seedlings: Data are presented as the mean ± standard deviation (*n* = 3), and significant differences between the control and endophytic YNA12 treatments were analyzed with one-way ANOVA followed by Duncan’s multiple range test (*p <* 0.05).

The effect of the 10-fold diluted culture of YNA12 was assessed to measure the catalase activity of evening primrose seedlings (Figure 4). The results demonstrated that the catalase activity of sole IAA-treated seedlings was significantly higher and that YNA12 culture broth-treated seedling exhibited significantly lower activity at 5 DAT. On the contrary, sole YNA12 culture broth-treated and combined YNA12 culture broth, ammonia, and IAA-treated seedling exhibited significantly higher CAT activity equally of CAT at 7 DAT as compared to those of sole IAA, ammonia-treated, and control seedlings, respectively.

### 3.6. Effect of YNA12 on the Regulation of the Endogenous ABA of Evening Primroses

**Figure 5:**
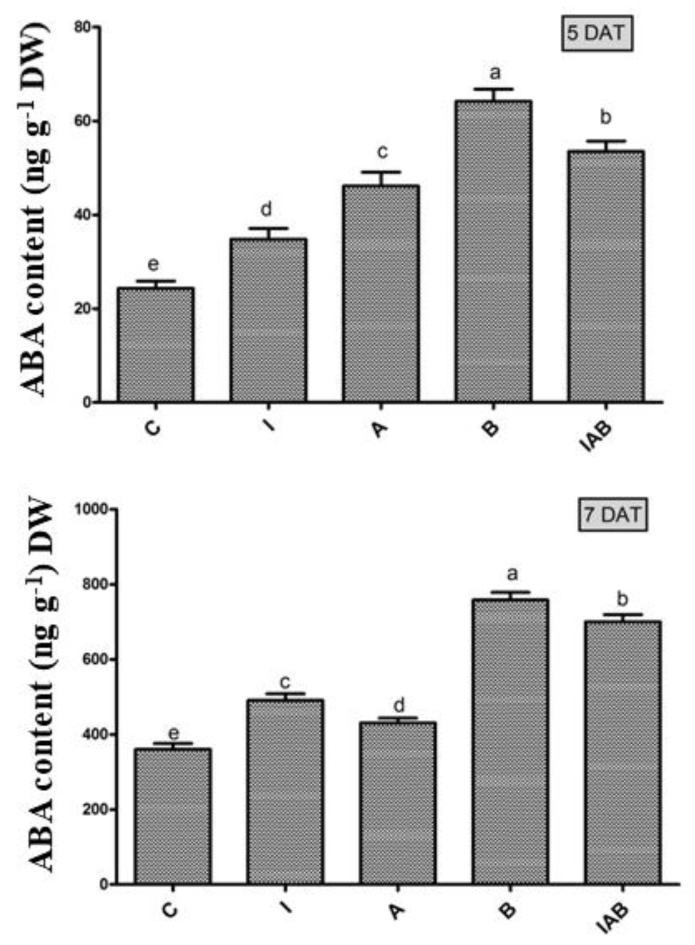
Effect of the YNA12 culture filtrate on the regulation of ABA in evening primrose seedlings: Data are presented as the mean ± standard deviation (*n* = 3), and significant differences between the control and endophytic YNA12 treatments were analyzed with one-way ANOVA followed by Duncan’s multiple range test (*p* < 0.05).

The regulation of endogenous ABA content has been reported to modulate seed dormancy and germination. In the current study, we investigated the effect of the culture broth of YNA12 on the ABA level of evening primroses at two different time points (5 DAT and 7 DAT). The results depicted that the ABA level of the YNA12 broth-treated seedling at 5 DAT was significantly higher by approximately 15%, 33%, 84%, and 164% as compared to those of combined treated, sole ammonia-treated, sole IAA-treated, and control seedlings, respectively (Figure 5), whereas the accumulation of ABA was significantly enhanced in evening primroses seedlings at 7 DAT. The findings illustrated that YNA 12 culture broth-treated seedlings exhibited significantly higher level (758.13 ng/g DW) of ABA, whilst control plants demonstrated a significantly lower level (384.3 ng/g DW) of ABA at 7 DAT (Figure 5).

### 3.7. Effect of YNA12 on the Macronutrient Regulation of Evening Primroses

**Figure 6:**
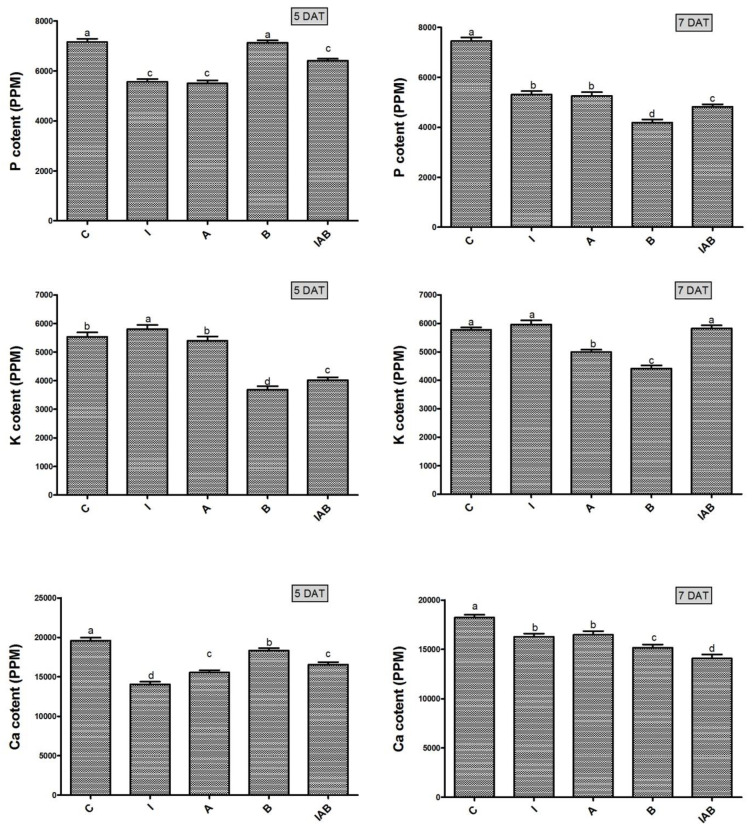
C, control seedlings; I, IAA-treated seedlings; A, ammonia-treated seedlings; B, YNA12-treated seedlings; and IAB, IAA-, ammonia-, and YNA12-treated seedlings. Different letters on the columns with error bars (mean ± standard deviation) represent significant differences among the treatments. The data were analyzed with a one-way ANOVA followed by Duncan multiple range test (*p* < 0.05).

The culture broth of YNA12 significantly modulated the endogenous macronutrients (P, K, and Ca) of evening primrose seeds (Figure 6). The level of P was not significantly different. The findings illustrated that the level of P was not significantly different at 5 DAT between YNA12-broth treated and control seedlings and exhibited significantly higher levels as compared to the combined treated and the sole IAA- and ammonia-treated seedlings. However, the level of P was significantly reduced in YNA12 broth-treated seedlings at 7 DAT, followed by those of the combined treated and the sole IAA- and ammonia-treated seedlings. On the other hand, the level of K in YNA12 broth-treated seedlings was significantly lower compared with control seedlings, combined treated seedlings, and sole IAA- and ammonia-treated seedlings at 5 DAT and 7 DAT. Similarly, a significantly higher amount of Ca at 5 DAT was observed in control seedlings followed by YNA12 broth treated-seedlings, sole ammonia, IAA-treated seedlings, and combined treated seedlings, respectively, whereas combined treated seedlings depicted significantly lower levels of Ca at 7 DAT followed by YNA12 broth-treated seedlings, sole ammonia seedlings, IAA-treated seedlings, and control seedlings, respectively.

## 4. Discussion

Together with the ability to promote host plant growth, endophytic microbes are also well documented for inducing deleterious effects on weed plants for inhibiting their growth and development [26]. Plant growth-promoting endophytes have been reported to exhibit host-specific behavior, acting as beneficial symbionts in some plants but as pathogens in others [27]. Moreover, the production of various metabolites by endophytes has been shown to have phytotoxic effects on non-host species. Controlling weed growth through endophytes has distinctive advantages over the application of pathogens, such as improving the ability of candidate microbes to survive in filed conditions via having consistent ecological niches within their host plant [26,28]. Therefore, the application of plant-promoting bacteria can be considered another environmentally sound technique for weed management as well as for reducing the application of synthetic fertilizers [28]. Moreover, the application of bacteria as biocontrol agents is considered more prevalent and selective as compared to synthetic herbicides by targeting only desired species and by lessening the chances of induction of resistance in target weeds due to employing various mechanisms for inhibiting their growth [28]. Furthermore, allelopathic compounds have been identified from endophytic microbes with a major role in weed management. Fourteen indole diketopiperazine (DKP) alkaloids, including spirotryprostatins (1–3), tryprostatins (4–6), and cyclotryprostatins (7–14), were obtained from endophytic *Aspergillus fumigatus* with significant plant growth-inhibiting potential [29].

Similarly, the inhibitory effect of culture filtrates of endophytic YNA12 on the growth of evening primrose seeds was assessed, which demonstrated adverse effects on the germination rate. The influence of the culture broth of YNA12 on inhibiting the germination rate of evening primroses was significantly promising as compared to sole IAA, ammonia-treated, and control seedlings. Our results demonstrated that the diluted culture broth (100 ppm) of YNA12 led to a significant reduction in seedling length and biomass. The inhibition of the germination rate, seedling length, and seedling biomass by the culture broth of YNA12 in the current study is in agreement with the findings of Barghouthi and Salman [30], who reported the germination inhibition and radical length reduction of *O. aegyptiaca* at higher concentrations using *Pseudomonas aeruginosa* QUBC1. Moreover, in the current study, the suppression of the germination of evening primroses by endophytic YNA12 may be attributed to the availability of various secondary metabolites in the culture broth, such as ammonia and IAA [7,31]. Previously, the emission of ammonia by *S. odorifera* 4Rx13 has been reported to significantly inhibit the growth of *A. thaliana* [31], whereas in the current study, we observed the germination inhibitory role of exogenous application of IAA and ammonia. Therefore, the inhibition of the germination rate as well as the seedling length and biomass by YNA12 can be attributed to its higher IAA-producing ability, which can exert negative effects on plant growth. In another study, the high concentration of IAA produced by *Pseudomonas* significantly inhibited durum wheat germination and growth rate [32]. Previously, Trognitz et al. also reported that rhizospheric or endophytic bacteria produce host-specific phytotoxic metabolites, such as IAA, in higher concentrations causing phytotoxicity by activating ethylene synthesis via induction of ACC synthase in shoot and production of abscisic acid and subsequently leading to the inhibition of plant growth [14]. Taken together, these findings suggest that endophytic YNA12 may inhibit the growth and development of unwanted weeds under normal conditions in an agricultural system.

To elucidate the mechanism underlying the detrimental effects of endophytic YNA12 on the germination of evening primroses, a biochemical analysis including the assessment of endogenous ABA regulation in germinated seeds was performed. Previously, high-level ABA has been reported as a potent inhibitor of seed germination by reducing the availability of energy and nutrients [33]. The results of our study demonstrated that the application of endophytic YNA12 broth resulted in a higher level of ABA accumulating in evening primrose seeds. The significant accumulation of ABA in the bacterial broth-treated seeds may have resulted in a marked reduction in gibberellins, leading to the retarded germination rate, seedling length, and seedling biomass. Previously, Radhakrishnan et al. [34] also reported that the application of *Enterobacter sp*. I-3 to weed seeds (*Echinochloa crus-galli* L. and *Portulaca oleracea* L.) significantly inhibited their germination via the accumulation of ABA and subsequently blocked GA biosynthetic pathways for controlling seedling growth. The suppression of evening primrose germination by YNA12, which increased the ABA level, may also be attributed to the reduced accumulation of calcium as a higher concentration of Ca^2+^ has been reported to counteract ABA effects by reducing *ABI4* expression during Ca^2+^ -dependent germination [35]. In addition, the ammonia-producing ability of YNA12 may have altered calcium homeostasis in evening primrose seedlings, resulting in the inhibition of seed germination. A higher level of ammonia in plant cells has been reported to exert adverse effects such as chlorosis, reduced root/shoot ratio, and inhibition of seedling establishment [31].

Similarly, the culture filtrate of YNA12 reduced the germination rate by affecting macronutrient (P, K, and Ca) regulation in evening primrose seeds. The current findings demonstrated the adverse effect of the culture filtrate of YNA12 on the nutrient uptake and regulation of evening primrose seedlings at two time points. The reduction in the nutrient uptake of evening primroses may be attributed to changes in the structure and function of the cellular membrane caused by the presence of allelochemicals in the culture filtrate of YNA12 [34]. Bacteria with allelopathic effects either directly influence plant growth by hampering metabolic and physiological processes through the release of allelochemicals or indirectly through the inhibition of an essential symbiont [36]. In the current study, the decrease in the macronutrient content of culture filtrate-treated seedlings may be attributed to the availability of excess ammonia, which has been reported to cause ionic imbalances in plants and to lead to nutritional deficiencies [37]. Therefore, the reduction in essential nutrients in YNA12 culture filtrate-treated evening primrose seeds resulted in germination inhibition and retarded seedling growth. However, the exact mechanism of nutrient uptake inhibition in evening primrose seedlings by the YNA12 culture filtrate requires further investigation at the transcriptomic and molecular levels.

## 5. Conclusions

The current study investigated the potential of endophytic *Klebsiella pneumoniae* YNA12 as a rich source of herbicidal metabolites. The culture filtrate of YNA12 contained IAA and ammonia and exerted adverse effects on evening primrose germination as well as seedling length and biomass. Moreover, the YNA12 culture filtrate increased the endogenous ABA level and suppressed the macronutrient uptake and antioxidant activity of evening primroses. Therefore, further large-scale studies are required to rectify the effectiveness of YNA12 application to agricultural fields as an alternative to chemical herbicides for inhibiting weed plant growth and development, whereas the emphasis should also be given to the extraction, isolation, and purification of YNA12 metabolites for producing vital compounds and subsequently their trails for inhibiting weed growth in agricultural fields.

## Figures and Tables

**Table 1:** Adverse effect of different treatments on the germination of evening primrose seeds.

Treatments	GR(%)		SL(cm)		SW(mg)		CDF(%)	
	5D	7D	5D	7D	5D	7D	5D	7D
Control	38.3 ± 3.5a	50.0 ± 8.66a	1.54 ± 0.09a	2.10 ± 0.27a	2.58 ± 0.28a	4.1 ± 0.8a	52.2	73.3
IAA 100 ppm	29.0 ± 4.0b	36.6 ± 2.88b	0.84 ± 0.05b	1.20 ± 0.13b	1.57 ± 0.30b	2.1 ± 0.22b	13.5	17.6
Ammonia 10 ppm	16.0 ± 1.7c	29.0 ± 1.73c	0.58 ± 0.08c	1.08 ± 0.18b	1.00 ± 0.04c	1.38 ± 0.13c	11.5	15.6
YNA 12 Culture Filtrate	11.0 ± 2.6d	22.3 ± 2.65d	0.30 ± 0.04d	0.40 ± 0.029c	0.84 ± 0.03d	1.12 ± 0.15c	5.2	13.6
A+I+YNA12	10.0 ± 1.0d	16.7 ± 1.53e	0.20 ± 0.04e	0.34 ± 0.041c	0.76 ± 0.05d	1.06 ± 0.14c	3.2	10.0

Means followed by different letters in the column are significantly different (*p* ≤ 0.05) as determined by Duncan’s multiple range test (DMRT): Each Petri dish contained 30 evening primrose seeds. GR, Germination rate; SL, Seedling length; SW, Seedling weight; and CDF, Cotyledon development frequency. D denotes the days after treatment.
